# Modeling Longitudinal Relationships Between CKD-MBD Biomarker Trajectories with Interpretable Machine Learning in a Large Prospective CKD Cohort

**DOI:** 10.3390/jcm15103690

**Published:** 2026-05-11

**Authors:** Tolgay Taskapan, Hulya Taskapan, Antonio Bellasi, Sara Mahdavi, Haley Ma, Paul Tam, Tabo Sikaneta

**Affiliations:** 1 Research Department, Kidney Life Sciences Institute, Toronto, ON M5G 2N2, Canada; taskapantolgay@gmail.com (T.T.); hy.haley.ma@gmail.com (H.M.);; 2 Department of Nephrology, University of Toronto, Toronto, ON M5S 3H2, Canada; 3 Nephrology Department, Ente Ospedaliero Cantonale, 6900 Lugano, Switzerland; antonio.bellasi@eoc.ch; 4 Department of Nutritional Science, Faculty of Medicine, University of Toronto, Toronto, ON M5S 1A1, Canada; sara.mahdavi@utoronto.ca; 5 Department of Nutrition, Harvard T.H. Chan School of Public Health, Harvard University, Boston, MA 02115, USA; 6 Nephrology Department, Scarborough Health Network, Toronto, ON M1P 2V5, Canada; 7 Department of Family and Community Medicine, University of Toronto, Toronto, ON M5S 1A1, Canada

**Keywords:** CKD-MBD, parathyroid hormone, FGF23, phosphate, calcium, longitudinal modeling, machine learning, SHAP

## Abstract

**Background/Objectives:** Chronic kidney disease–mineral and bone disorder (CKD-MBD) is characterized by complex interactions between parathyroid hormone (PTH), fibroblast growth factor 23 (FGF23), phosphate, calcium, and vitamin D, yet clinical decisions are often based on static individual values. A trajectory-based approach that considers how longitudinal changes in CKD-MBD biomarkers relate to each other may provide a more integrated understanding. **Methods**: In 1968 adults with non-dialysis CKD (mean age 68 ± 12 years; 34% female; mean follow-up duration 3.02 ± 0.35 years), annualized slopes of CKD-MBD biomarkers (PTH, FGF23, phosphate, calcium) were derived from mixed-effects models, alongside slopes and intra-individual variability of other key features of CKD—eGFR, hemoglobin, albumin, bicarbonate, 25-hydroxyvitamin D, uric acid, urine albumin creatinine ratio (UACR). These 36 features, including baseline levels, were evaluated using Extreme Gradient Boosting regression with interpretability via SHapley Additive exPlanations (SHAP). **Results**: Models explained substantial variance in biomarker slopes (R^2^: 0.86 for PTH, 0.72 for FGF23, 0.71 for phosphate, 0.67 for calcium) without evidence of overfitting. Baseline values and eGFR slopes showed the strongest associations with biomarker trajectories. PTH and FGF23 slopes were positively correlated, while declining hemoglobin and greater hemoglobin variability were associated with steeper FGF23 and phosphate slopes. Phosphate slopes were associated with UACR slopes and bicarbonate slopes. Declining 25(OH)D slopes were linked with rising PTH slopes. **Conclusions**: This analysis revealed consistent relationships between CKD-MBD biomarker trajectories. Longitudinal changes in kidney function, hemoglobin, bicarbonate, UACR, and vitamin D were also associated with biomarker trajectories. Our findings confirm the multi-system nature of CKD-MBD, and provide a framework for examining potentially modifiable pathways in its progression.

## 1. Introduction

Chronic kidney disease–mineral and bone disorder (CKD-MBD) is a multisystem complication of CKD characterized by dysregulation of parathyroid hormone (PTH), fibroblast growth factor 23 (FGF23), phosphate (PO4), calcium, and vitamin D. These abnormalities contribute to skeletal disease, vascular calcification, CKD progression, cardiovascular events, and excess mortality [1,2,3,4,5,6]. Despite its clinical significance, optimal assessment and management remain challenging.

Current guidelines [2,7] emphasize recognizing patients with persistently elevated or progressively rising PTH, and interpreting PTH in conjunction with calcium, phosphate, and vitamin D. However, in clinical practice decisions are often based on intermittent, static laboratory values. This “snapshot” approach overlooks dynamic biomarker trajectories, delaying recognition of progressive biochemical abnormalities that may have prognostic and therapeutic importance. Longitudinal analyses offer a complementary perspective by quantifying trajectories—the annualized slope and intra-individual variability of biomarkers—rather than relying solely on absolute concentrations. Prior studies have shown that elevated baseline levels of PTH, phosphate, or FGF23 are associated with adverse outcomes and that kidney function decline drives worsening mineral profiles [1,2,3,4,5,6,8]. Far less is known, however, about how these trajectories relate to each other—for example, how PTH changes in parallel with FGF23 or vitamin D, or how changes in non-CKD-MBD features such as hemoglobin, albumin, and bicarbonate shape mineral metabolism over time. Addressing this gap may clarify the multisystem cross-talk that characterizes CKD-MBD.

Advances in explainable artificial intelligence permit machine learning models to move beyond risk prediction and contribute to understanding of disease biology. SHapley Additive exPlanations (SHAP) [9] quantify the relative influence of kidney function, mineral markers, and systemic features on biomarker dynamics, offering a transparent bridge between statistical learning and CKD-MBD pathophysiology. Accordingly, we applied interpretable machine learning to a large prospective CKD cohort with repeated longitudinal measurements. Extreme Gradient Boosting (XGBoost) was selected as the machine learning algorithm due to its proven efficacy in handling complex datasets with mixed data types, its robustness to outliers, and its ability to capture non-linear relationships and interactions among predictors without explicit prior specification. Furthermore, its inherent feature importance capabilities align well with the need for interpretability in clinical research [10]. Our objectives were: (1) to identify the strongest determinants of trajectories of PTH, FGF23, calcium and phosphate (2) to assess the relative contributions of baseline values, kidney decline, and variability in other features of CKD; and (3) to use SHAP to reveal physiologically meaningful inter-relationships between mineral metabolism, hematologic, nutritional, and renal pathways.

## 2. Materials and Methods

### 2.1. Study Design and Population

This study used data from the CAN-AIM to PREVENT Trial, a prospective, observational cohort study conducted in Toronto, Ontario, Canada, between 2010 and 2015 (ClinicalTrials.gov Identifier: NCT01974713). The original trial followed 2254 patients with pre-dialysis CKD clinics to identify predictive markers for progression to dialysis. Eligible participants for the original trial were aged 18 years or older, had an estimated glomerular filtration rate (eGFR) < 60 mL/min/1.73 m^2^, and were either under the care of a nephrologist or awaiting a nephrology consultation. Key exclusion criteria included active renal replacement therapy (including prior organ transplantation), a projected life expectancy of less than 12 months, the use of or contraindications to an erythropoietin-stimulating agent, and an anticipated need for renal replacement therapy within six months of enrollment. This study was conducted in strict accordance with the ethical principles outlined in the Declaration of Helsinki. Ethical approval for the research protocol was obtained prior to participant recruitment, and written informed consent was obtained from all individual participants included in the study. We included a subset of 1968 patients with a baseline estimated glomerular filtration rate (eGFR) greater than 15 and up to 59 mL/min/1.73 m^2^.

### 2.2. Data Collection

Demographic, clinical, and laboratory variables were prospectively collected every six months across the 3-year follow-up period. Laboratory tests included serum creatinine used to calculate eGFR with the 2009 CKD-EPI equation [11], albumin, calcium, phosphate, bicarbonate, uric acid, hemoglobin, 25-hydroxyvitamin D (25(OH)D), PTH, and FGF23, as well as urine albumin-to-creatinine ratio (UACR). Medication use (calcium-based binders and vitamin D analogs) and comorbidities (e.g., diabetes) were also recorded.

### 2.3. Statistics

Prior to analysis, variables with right-skewed distributions (PTH, FGF23, 25(OH)D, uric acid and UACR) were natural log-transformed to stabilize variance and approximate normality. Missing covariate data, which accounted for <10% of all observations, were handled using multiple imputation by chained equations (MICE), generating 20 complete datasets under the missing-at-random assumption. MICE was selected because it iteratively imputes missing variables using robust regression models conditional on all other available data. This approach preserves statistical power, properly accounts for imputation uncertainty, and significantly reduces selection bias compared to traditional methods such as complete-case analysis or single mean imputation.

The primary outcomes were the annualized slopes of four key CKD-MBD biomarkers: phosphate, calcium, log-transformed PTH, and log-transformed FGF23. To capture broader longitudinal disease dynamics, annualized slopes were also estimated for eGFR, hemoglobin, albumin, bicarbonate, log-transformed 25(OH)D, log-transformed uric acid, and log-transformed UACR. For each participant, these slopes were derived from linear mixed-effects models with a fixed effect for time and random intercepts and slopes, allowing estimation of both population-level trends and individual-level trajectories. Within-person standard deviations (SD) of these longitudinal measures were also calculated and used as indicators of temporal variability. These slope and variability estimates were subsequently used as explanatory features in the machine learning analyses.

Annualized slopes of four key CKD–MBD biomarkers, log-PTH, log-FGF23, serum calcium, and phosphate, served as primary outcomes. To characterize broader disease dynamics, additional slopes were estimated for eGFR, hemoglobin, albumin, bicarbonate, log-25(OH)D, log-uric acid, and log-UACR. Each participant’s slope was derived from a linear mixed-effects model with a fixed effect for time and random intercepts and slopes, providing both population-level trends and within-person variability. These auxiliary trajectories were subsequently used as explanatory features in later modeling.

A fixed set of the explanatory variables was then defined for each participant, combining:

1. Baseline characteristics: age, sex, diabetes history, calcium-based drug use, vitamin D use, and baseline laboratory values (eGFR, calcium, phosphate, albumin, bicarbonate, log uric acid, and hemoglobin, log UACR, log 25(OH)D, log PTH, log FGF23).
2. Dynamic features: annualized slopes and within-person standard deviations (SDs) of clinically relevant measures (eGFR, hemoglobin, albumin, bicarbonate, phosphate, calcium, log uric acid, log UACR, log PTH, log FGF23, and log 25(OH)D).

Four separate explanatory machine learning models were then fitted using XGBoostRegressor 3.0.5, with the annualized slope of phosphate, calcium, log-transformed PTH, or log-transformed FGF23 specified as the outcome in turn. The same baseline characteristics was included in all four models. Dynamic features were incorporated according to a prespecified rule. When a given biomarker slope was the outcome, that biomarker’s baseline value was included in the model to account for initial status, whereas its own within-person SD was excluded to avoid self-correlation. In contrast, the slopes and within-person SDs of the remaining longitudinal biomarkers were included as predictors. This standardized, rule-based specification was applied uniformly across all four models to ensure consistency, improve comparability, and reduce the risk of circular inference.

Data for each biomarker model were randomly partitioned into training (70%) and testing (30%) sets. Five-fold cross-validation within the training data guided hyperparameter tuning and internal model evaluation. Analyses were repeated across all imputed datasets, and pooled results reflected model stability and uncertainty. To test robustness and mitigate spurious associations, 1000-iteration permutation tests were performed per outcome. Model performance was summarized using the coefficient of determination (R^2^) and root-mean-square error (RMSE) on the test set, with 95% confidence intervals obtained via bootstrap resampling.

Model interpretability was achieved using SHapley Additive exPlanations (SHAP) [9], an interpretable machine learning framework that decomposes model predictions into additive contributions from individual predictors. Based on cooperative game theory, SHAP quantifies the average marginal contribution of each variable across all possible model combinations. SHAP values were used to identify and visualize the strength, direction, and nonlinearity of associations between systemic predictors and biomarker trajectory slopes. SHAP summary plots were averaged across imputations, while dependence plots explored individualized and nonlinear feature effects. These analyses identified the most influential predictors of biomarker trajectories, clarifying their roles in dynamic CKD-MBD progression.

The overall study design is shown in Supplementary Figure S1. All mixed-effects slope models and descriptive statistics were performed using Stata Statistical Software (Release 19; StataCorp, College Station, TX, USA, 2025), while all machine learning analyses and SHAP visualizations were conducted in Python 3.13.

## 3. Results

### 3.1. Cohort Characteristics

We analyzed 1968 adults with CKD (mean age 68 ± 12 years; 34% female; 47% diabetes). Mean baseline eGFR was 41 ± 13 mL/min/1.73 m^2^. The mean follow-up duration was 3.02 ± 0.35 years, yielding 11,354 observations. Baseline demographics and laboratory results are shown in Table 1, while longitudinal slopes and intra-individual variability are summarized in Table 2. The study flow and cohort selection are summarized in Supplementary Figure S1.

### 3.2. Model Performance

The SHAP analysis applied to the XGBoost models demonstrated consistently strong and stable predictive performance across all biomarker trajectories (Table 3). Cross-validated coefficients of determination (R^2^) were 0.86 ± 0.01 for the log PTH slope, 0.72 ± 0.02 for the log FGF23 slope, 0.67 ± 0.05 for the phosphate slope, and 0.67 ± 0.02 for the calcium slope. Corresponding root-mean-square errors (RMSE ± SD) were 0.14 ± 0.01, 0.21 ± 0.02, 0.076 ± 0.01, and 0.048 ± 0.005, respectively, indicating small residual variance and reliable model calibration. All permutation tests produced significant results (*p* < 0.01), confirming that each model captured genuine, non-random predictive structure. Learning curves exhibited nearly parallel declines in training and validation RMSE that plateaued around 80 boosting iterations, demonstrating strong regularization and the absence of overfitting (Supplementary Figures S2–S5).

### 3.3. Phosphate Trajectories

**Figure 1:**
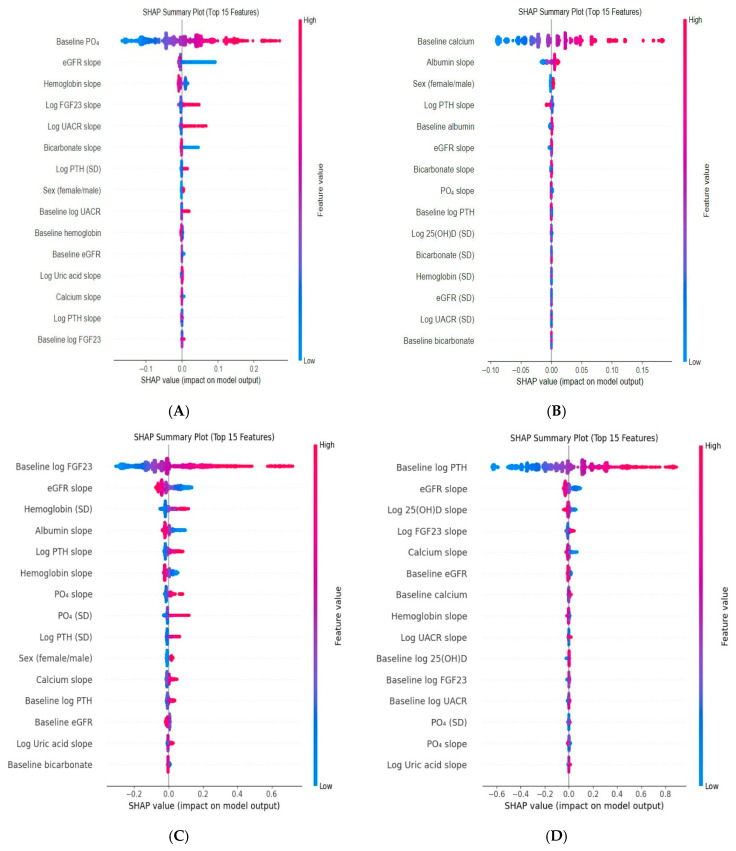
SHAP summary plot of the top 15 features ranked by mean absolute contribution to the model output (**A**). PO4 slope, (**B**). Calcium slope, (**C**). Log FGF23 slope, (**D**). Log PTH slope. Within each panel, features are ordered on the y-axis by mean absolute SHAP value, which represents overall feature importance, whereas the x-axis displays the SHAP value, reflecting the direction and magnitude of each feature’s effect on the model output. Abbreviations: eGFR: Estimated Glomerular Filtration Rate; 25(OH)D: 25-hydroxyvitamin D; FGF23: Fibroblast Growth Factor 23; PO_4_: Phosphate; PTH: Parathyroid Hormone; SD: Standard Deviation; UACR: Urine Albumin-to-Creatinine Ratio; log: natural logarithm (base e).

**Figure 2:**
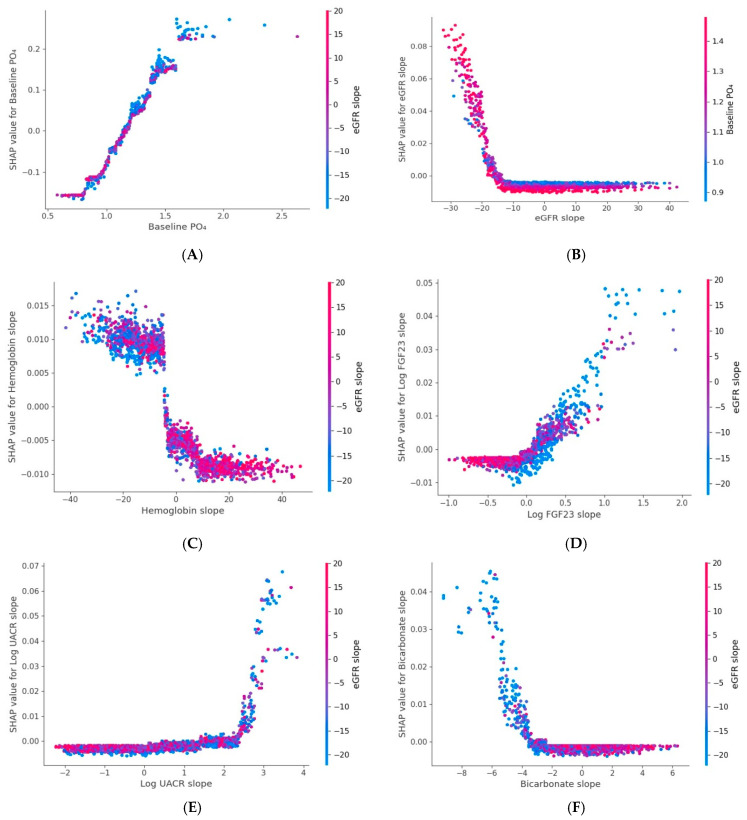
SHAP dependence plots for six key variables associated with phosphate slope model. (**A**). Baseline PO_4_; (**B**). eGFR slope; (**C**). Hemoglobin slope; (**D**). Log-FGF23 slope; (**E**). Log-UACR slope; (**F**). Bicarbonate slope. Each dot represents an observation. The x-axis shows the feature value, and the y-axis displays its SHAP value, indicating its contribution (direction and magnitude) to the predicted phosphate slope. Dot color reflects an interacting feature’s value, with the color bar showing its range. Positive SHAP values denote a positive contribution, while negative values denote a negative contribution. Abbreviations: eGFR: Estimated Glomerular Filtration Rate; FGF23: Fibroblast Growth Factor 23; PO_4_: Phosphate; UACR: Urine Albumin-to-Creatinine Ratio; log: natural logarithm (base e).

SHAP analysis of the phosphate slope model identified six key variables, baseline phosphate, eGFR slope, hemoglobin slope, log FGF-23 slope, log UACR slope, and bicarbonate slope, as the strongest associates of phosphate change. Dependence plots showed that faster eGFR decline and higher baseline phosphate predicted steeper phosphate increases. Declining hemoglobin slope was negatively related to phosphate change, whereas increases in log FGF-23 slope, log UACR slope, and decreases in bicarbonate were linked with greater phosphate rises (Figure 1 and Figure 2).

### 3.4. Calcium Trajectories

**Figure 3:**
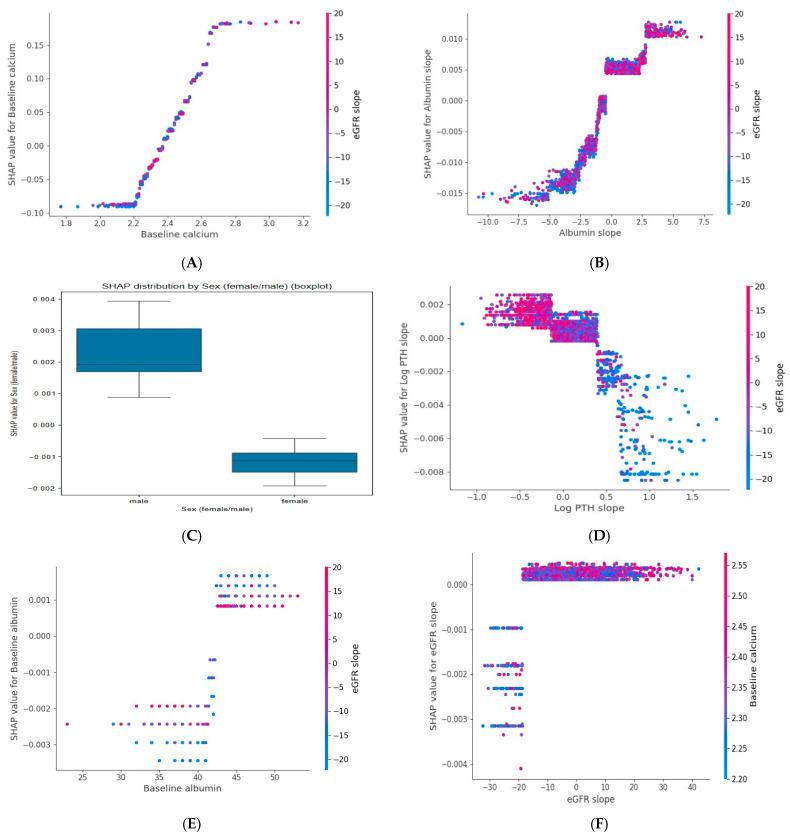
SHAP dependence plots for six key variables associated with calcium slope model (**A**). Baseline calcium, (**B**). Albumin slope (**C**). Sex, (**D**). PTH slope, (**E**). Baseline albumin and (**F**). Baseline eGFR. Each dot represents an observation. The x-axis shows the feature value, and the y-axis displays its SHAP value, indicating its contribution (direction and magnitude) to the predicted calcium slope. Dot color reflects an interacting feature’s value, with the color bar showing its range. Positive SHAP values denote a positive contribution, while negative values denote a negative contribution. Abbreviations: eGFR: Estimated Glomerular Filtration Rate; PTH: Parathyroid Hormone; log: natural logarithm (base e).

According to SHAP analysis of the calcium model, six principal variables, baseline calcium, albumin slope, sex, baseline albumin, PTH slope, and eGFR slope, were most associated with calcium slope. Baseline calcium, albumin slope, male sex, and baseline albumin showed positive association with calcium slope, whereas PTH slope and eGFR slope exhibited negative associations (Figure 1 and Figure 3).

### 3.5. FGF-23 Trajectories

**Figure 4:**
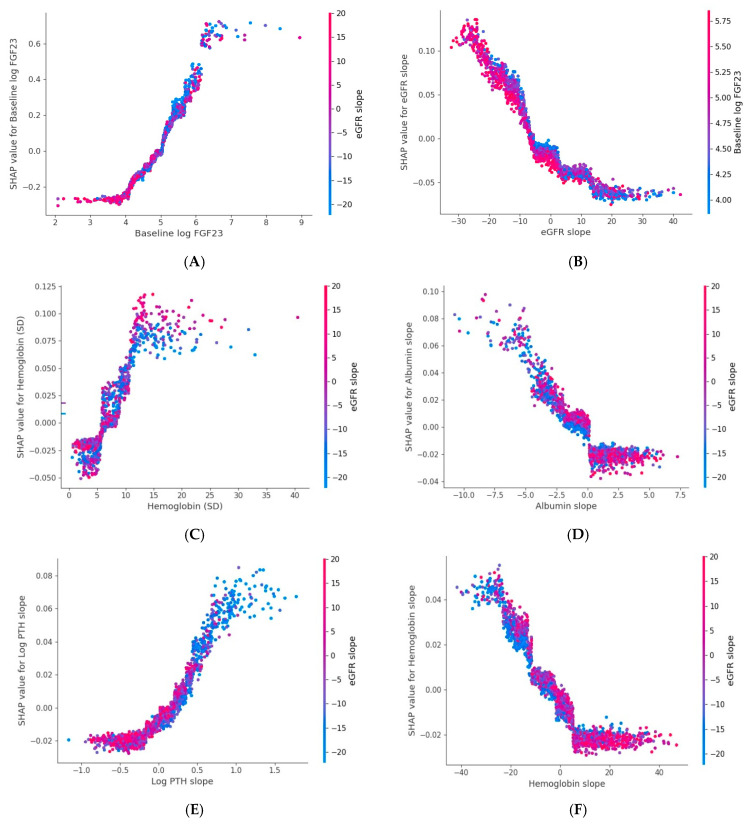
SHAP dependence plots for six key variables associated with log FGF23 slope model (**A**). Baseline log-FGF23, (**B**). Baseline eGFR (**C**). Hemoglobin variability, (**D**). Albumin slope, (**E**). Log-PTH slope and (**F**). Hemoglobin slope. Each dot represents an observation. The x-axis shows the feature value, and the y-axis displays its SHAP value, indicating its contribution (direction and magnitude) to the predicted log FGF23 slope. Dot color reflects an interacting feature’s value, with the color bar showing its range. Positive SHAP values denote a positive contribution, while negative values denote a negative contribution. Abbreviations: eGFR: Estimated Glomerular Filtration Rate; FGF23: Fibroblast Growth Factor 23; PTH: Parathyroid Hormone; SD: Standard Deviation; log: natural logarithm (base e).

SHAP analysis of the log FGF-23 model highlighted six main variables: baseline log FGF-23, eGFR slope, hemoglobin variability, albumin slope, log PTH slope, and hemoglobin slope. Dependence plots demonstrated that faster eGFR decline and greater hemoglobin variability were associated with steeper log FGF-23 increases, while declining albumin and hemoglobin slopes were both linked to higher FGF-23 slopes. A positive correlation between log FGF-23 and log PTH slopes was also evident (Figure 1 and Figure 4).

### 3.6. PTH Trajectories

**Figure 5:**
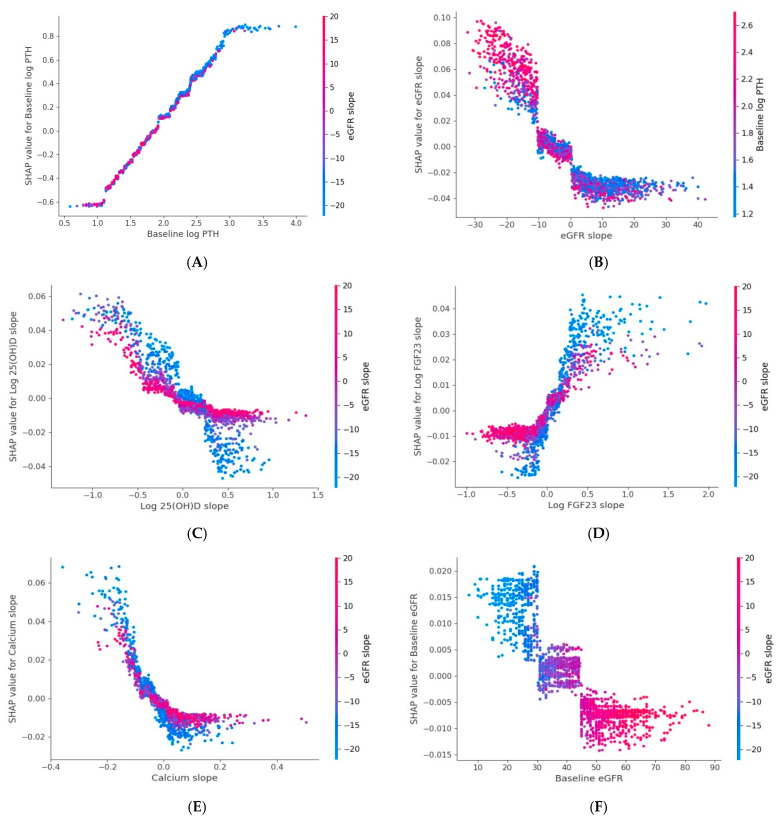
SHAP dependence plots for six key variables associated with log PTH slope model (**A**). Baseline log-PTH, (**B**). Baseline eGFR (**C**). 25(OH)D slope, (**D**). Log-FGF23 slope, (**E**). Calcium slope and (**F**). Baseline eGFR. Each dot represents an observation. The x-axis shows the feature value, and the y-axis displays its SHAP value, indicating its contribution (direction and magnitude) to the predicted log PTH slope. Dot color reflects an interacting feature’s value, with the color bar showing its range. Positive SHAP values denote a positive contribution, while negative values denote a negative contribution. Abbreviations: eGFR: Estimated Glomerular Filtration Rate; 25(OH)D: 25-hydroxyvitamin D; FGF23: Fibroblast Growth Factor 23; PTH: Parathyroid Hormone; log: natural logarithm (base e).

SHAP analysis identified six key predictors of PTH slope; baseline PTH, eGFR slope, log 25(OH)D slope, log FGF-23 slope, calcium slope, and baseline eGFR. Steeper PTH increases were associated with higher baseline PTH and faster eGFR decline. Dependence plots further showed that a declining log 25(OH)D slope was associated with an accelerated rise in PTH, particularly in the context of concurrent kidney function decline (Figure 1 and Figure 5).

Supplementary Figure S6 presents SHAP analysis identifying the top 15 features contributing to the prediction of PO_4_, calcium, Log FGF23, and Log PTH slopes.3.2.

## 4. Discussion

In this study, we evaluated associations between longitudinal trajectories of key CKD-MBD biomarkers (PTH, FGF23, and phosphate) and other parameters of CKD using interpretable machine learning applied to a large prospective CKD cohort. Our approach incorporated baseline levels, variability, and slopes of these biomarkers, and allowed us to identify consistent patterns of associations across mineral, renal, hematologic, and metabolic domains. Three main findings emerged: (i) baseline biomarker levels were strongly associated with subsequent slopes, (ii) decline in kidney function was consistently associated with worsening trajectories, and (iii) non-traditional factors such as slopes of hemoglobin, albumin, urine protein, and bicarbonate showed independent associations with biomarker slopes.

Baseline levels of PTH, FGF23, and phosphate had the strongest associations with their respective slopes. Patients with higher baseline concentrations tended to show steeper subsequent increases, an observation that supports the notion of “biomarker momentum.” This finding is consistent with earlier cohort work demonstrating that once abnormalities in mineral metabolism appear, they often persist and intensify [1,12]. Our results suggest that elevated baseline values may mark individuals at higher risk for progressive biochemical changes, although they should be understood primarily as indicators of disease burden and prior exposures rather than causal factors.

Declining kidney function, summarized as eGFR slope, was strongly associated with the worsening of all three biomarkers. Faster eGFR loss was related to higher rates of increase in PTH, FGF23, and phosphate, and decrease in calcium echoing longstanding evidence that kidney decline is closely tied to disturbances in mineral metabolism [1,13]. However, it also suggests that these metabolic abnormalities might further accelerate CKD progression and underscore the importance of conducting additional interventional trials to evaluate how CKD-MBD modulation affects the course of CKD. In addition, worsening albuminuria was associated specifically with steeper phosphate slopes, indicating that proteinuria and glomerular injury may provide additional signals of altered phosphate handling [14]. Together, these findings reinforce the close link between renal deterioration and the trajectory of mineral metabolism abnormalities in CKD.

We observed a positive association between PTH and FGF23 slopes. While FGF23 normally suppresses PTH secretion and PTH stimulates FGF23 production [15,16,17,18], CKD is characterized by disruption of these regulatory loops [18,19,20,21,22]. The parallel increases in our cohort suggest that endocrine resistance and parathyroid hyperplasia may attenuate normal feedback, resulting in simultaneous upward trajectories. Our results therefore align with the concept of reciprocal dysregulation, though the direction of influence cannot be determined from these associations.

Vitamin D slopes also showed clear associations with parathyroid trajectories. Declining 25(OH)D slopes were correlated with steeper rises in PTH, consistent with the pathophysiology of secondary hyperparathyroidism, where reduced vitamin D availability impairs calcium absorption and stimulates parathyroid hormone secretion [23]. The dependence plots further indicated that this association was accentuated in patients with faster eGFR decline, highlighting an interaction between kidney function deterioration and vitamin D metabolism [24]. These results underscore the importance of considering not only baseline 25(OH)D levels but also their longitudinal trend, as progressive vitamin D decreasing may signal greater risk for worsening parathyroid activity. Clinically, this supports the potential adjunctive role of vitamin D repletion strategies as part of broader CKD management.

Calcium and PTH slopes showed inverse associations, consistent with expected negative feedback. However, the magnitude of this inverse relationship appeared attenuated at lower eGFR, suggesting possible partial uncoupling of the calcium–PTH axis in moderate CKD. This pattern is consistent with prior evidence that reduced renal function may diminish calcium-sensing receptor activity and calcitriol synthesis, potentially weakening feedback control [25,26,27,28]. Experimental studies indicate that PTH normally enhances urinary phosphate excretion and calcium retention [29], while CKD-related down-regulation of nephron pathways and reduced FGF-23/Klotho signaling could contribute to feedback impairment [30]. Together, these findings are compatible with, but do not prove, weakening of physiological feedback control during CKD progression.

Beyond renal decline, biomarker trajectories were also associated with systemic parameters. Declining hemoglobin slopes and greater within-person hemoglobin variability were statistically associated with steeper phosphate and FGF-23 slopes. While these associations are observational, previous experimental and clinical studies have described bidirectional links between anemia, disordered iron handling, phosphate retention, and FGF23 dysregulation [31,32,33,34,35,36,37,38], often conceptualized within a multi-component kidney-bone marrow-bone axis [22]. For example, phosphate loading and elevated FGF23 can impair erythropoiesis [37,38], while iron deficiency and anemia aggravate FGF23 abnormalities [34]. Future work should test whether improving hematologic parameters modifies mineral biomarker trajectories.

Similarly, declining albumin slopes were associated with rising FGF23 slopes, a finding that may be compatible with an influence of nutritional status on FGF23 dynamics [39]. Declining bicarbonate slopes were likewise associated with increasing phosphate slopes, which is biologically plausible given the known links between metabolic acidosis, bone buffering, and disordered mineral metabolism in CKD [40,41]. These associations demonstrate that CKD-MBD progression is shaped by multiple systemic domains and not only mineral metabolism itself.

Our findings highlight the value of examining biomarker trajectories rather than isolated static measurements. Patients with higher baseline values and steeper kidney function decline were more likely to show worsening slopes, indicating an opportunity for earlier risk identification. The associations with hemoglobin, albumin, bicarbonate, and vitamin D suggest that management of anemia, nutritional decline, metabolic acidosis, and vitamin D insufficiency may be relevant not only for general CKD health but also for moderating mineral metabolism abnormalities. Importantly, these observations should be viewed as hypothesis-generating, with future intervention studies required to test whether modifying these domains can alter biomarker trajectories or clinical outcomes.

Key strengths of this study include use of a large, longitudinally followed CKD cohort, robust slope estimation via mixed-effects models, and the application of interpretable machine learning to clarify associations. This approach provided flexible modeling of nonlinear effects and interactions while maintaining interpretability. The good predictive performance (R^2^ 0.67–0.86), the robustness of the methodology involving XGBoost and SHAP interpretability, along with the biological plausibility, are the key strengths of these findings.

Limitations include that exposures and outcomes derived from the same time window limit causal inference. Slopes were assumed to be linear, which may underestimate acceleration in later CKD. Data on diet, and iron status were unavailable or not analyzed and may have contributed to observed associations. Finally, replication of these findings in other CKD populations is warranted to increase generalizability.

## 5. Conclusions

In summary, interpretable machine learning applied to longitudinal CKD data revealed consistent associations between CKD-MBD biomarker trajectories. Associations of biomarker slopes with concurrent changes in hemoglobin, albumin, bicarbonate, and albuminuria indicate that mineral metabolism evolves in concert with other abnormalities seen in CKD. These findings emphasize the interconnected nature of CKD-MBD and illustrate the value of trajectory-based analyses for uncovering integrated patterns that merit further mechanistic and interventional investigation. Further validation and outcome studies are essential before trajectory-based monitoring can be recommended for practice.

## Figures and Tables

**Table 1:** Baseline characteristics of the study population.

Characteristic	Value
Age, years, mean (SD)	68.4 ± 12.2
Sex, female (%)	666 (33.8%)
Diabetes history, Yes (%)	927 (47.1%)
Calcium-based drug use, Yes (%)	45 (2.3%)
Vitamin D use, Yes (%)	447 (22.7%)
Hemoglobin, g/L, mean (SD)	129.73 ± 16.64
eGFR, mL/min/1.73 m^2^, mean (SD)	41.5 ± 13.3
UACR, mg/mmol, median (IQR)	7.9 (1.7–42.1)
Calcium, mmol/L, mean (SD)	2.37 ± 0.12
PO_4_, mmol/L, mean (SD)	1.16 ± 0.19
Albumin, g/L, mean (SD)	43.5 ± 3.1
Bicarbonate, mmol/L, mean (SD)	25.5 ± 3.2
Uric acid, µmol/L, median (IQR)	440.8 ± 109.2
25(OH)D, nmol/L, median (IQR)	65.0 (45.0–85.0)
PTH, pmol/L, median (IQR)	5.0 (3.5–7.6)
FGF23, pg/mL, median (IQR)	123.0 (85.1–168.7)

Abbreviations: eGFR: estimated glomerular filtration rate; UACR: urine albumin-to-creatinine ratio; PO_4_: phosphate; 25(OH)D: 25-hydroxyvitamin D; PTH: parathyroid hormone; FGF23: fibroblast growth factor-23; log: natural logarithm (base e); SD: Standard Deviation, IQR: Interquartile Range.

**Table 2:** Longitudinal slopes (annual change) and intra-individual variability across 20 imputed datasets.

Variable	Annually Slopes Value (Mean ± SD)	Variability (SD) Value (Mean ± SD)
Hemoglobin (g/L)	−0.85 ± 15.07	6.37 ± 3.93
eGFR (mL/min/1.73 m^2^/year)	−1.32 ± 12.86	5.02 ± 2.99
Log UACR (log-unit)	0.10 ± 1.34	54.10 ± 38.38
Calcium (mmol/L)	−0.005 ± 0.083	0.0073 ± 0.038
PO_4_ (mmol/L)	0.01 ± 0.13	0.13 ± 0.09
Albumin (g/L)	−0.28 ± 2.44	1.59 ± 0.80
Bicarbonate (mmol/L)	−0.34 ± 2.26	1.99 ± 0.84
Log uric acid (log-unit)	−0.02 ± 0.21	0.72 ± 0.43
Log 25(OH)D (log-unit)	0.08 ± 0.38	0.23 ± 0.15
Log PTH (log-unit)	0.03 ± 0.42	0.22 ± 0.14
Log FGF23 (log-unit)	−0.06 ± 0.36	0.46 ± 0.22

The values represent pooled estimates across 20 multiply imputed datasets slope values indicate the rate of change annually for each variable. SD: Standard deviation values reflect the variability of each measure. Abbreviations: eGFR: estimated glomerular filtration rate; PTH: parathyroid hormone; UACR: urine albumin-to-creatinine ratio; 25(OH)D: 25-hydroxyvitamin D; PO_4_: phosphate; FGF23: fibroblast growth factor-23; SD: standard deviation; log: natural logarithm (base e).

**Table 3:** Performance metrics of XGBoost models predicting biomarker slopes.

Biomarker Model	Mean R^2^ ± SD	95% CI for R^2^	RMSE ± SD	95% CI for RMSE	Permutation *p*
Calcium slope	0.67 ± 0.02	[0.64–0.69]	0.048 ± 0.005	[0.044–0.054]	<0.001
Phosphate slope	0.67 ± 0.05	[0.60–0.75]	0.076 ± 0.01	[0.067–0.087]	<0.001
Log FGF23 slope	0.72 ± 0.02	[0.70–0.74]	0.21 ± 0.02	[0.18–0.23]	<0.001
Log PTH slope	0.86 ± 0.01	[0.84–0.87]	0.14 ± 0.010	[0.13–0.16]	0.005

Each slope model estimates the longitudinal rate of change in the respective serum marker. Metrics represent pooled cross-validation results across multiply imputed datasets. Abbreviations: R^2^: coefficient of determination; RMSE: root-mean-square error; CI: bootstrap confidence interval; FGF23: Fibroblast growth factor; PTH: Parathyroid hormone; Log: natural logarithm (base e).

## Data Availability

The data that support the findings of this study are available from the corresponding author, [T.S.], upon reasonable request.

## Supplementary Materials

The following supporting information can be downloaded at: https://www.mdpi.com/article/10.3390/jcm15103690/s1, Figure S1. Analytic Workflow for Machine Learning Models and SHAP Interpretability in the CAN-AIM Cohort, Figure S2. Gradient boosting model performance for predicting phosphate (PO_4_) slope, Figure S3. Gradient boosting model performance for predicting calcium slope, Figure S4. Gradient boosting model performance for predicting log-FGF23 slope, Figure S5. Gradient boosting model performance for predicting log-PTH slope. Figure S6. SHAP bar plot of the top 15 features ranked by mean absolute contribution to the model output.

## Abbreviations

25(OH)D	25-hydroxyvitamin D
Ca	Calcium
CI	Confidence Interval
CKD	Chronic Kidney Disease
CKD-EPI	Chronic Kidney Disease Epidemiology Collaboration
CKD-MBD	Chronic Kidney Disease–Mineral and Bone Disorder
eGFR	Estimated Glomerular Filtration Rate
FGF23	Fibroblast Growth Factor 23
IQR	Interquartile Range
log	Natural logarithm
MICE	Multiple Imputation by Chained Equations
PO_4_	Phosphate
PTH	Parathyroid Hormone
R^2^	Coefficient of Determination
RMSE	Root Mean Square Error
SD	Standard Deviation
SHAP	SHapley Additive exPlanations
UACR	Urine Albumin-to-Creatinine Ratio
XGBoost	Extreme Gradient Boosting
