# Supplementary material for: Modeling Longitudinal Relationships Between CKD-MBD Biomarker Trajectories with Interpretable Machine Learning in a Large Prospective CKD Cohort
Source: J Clin Med. 2026 May 11;15(10):3690. doi: 10.3390/jcm15103690 (PMC13207337; doi:10.3390/jcm15103690)
Supplement: Supplementary file 1 [file jcm-15-03690-s001.zip › jcm-4241602-supplementary.pdf]

Journal of Clinical Medicine

MDPI

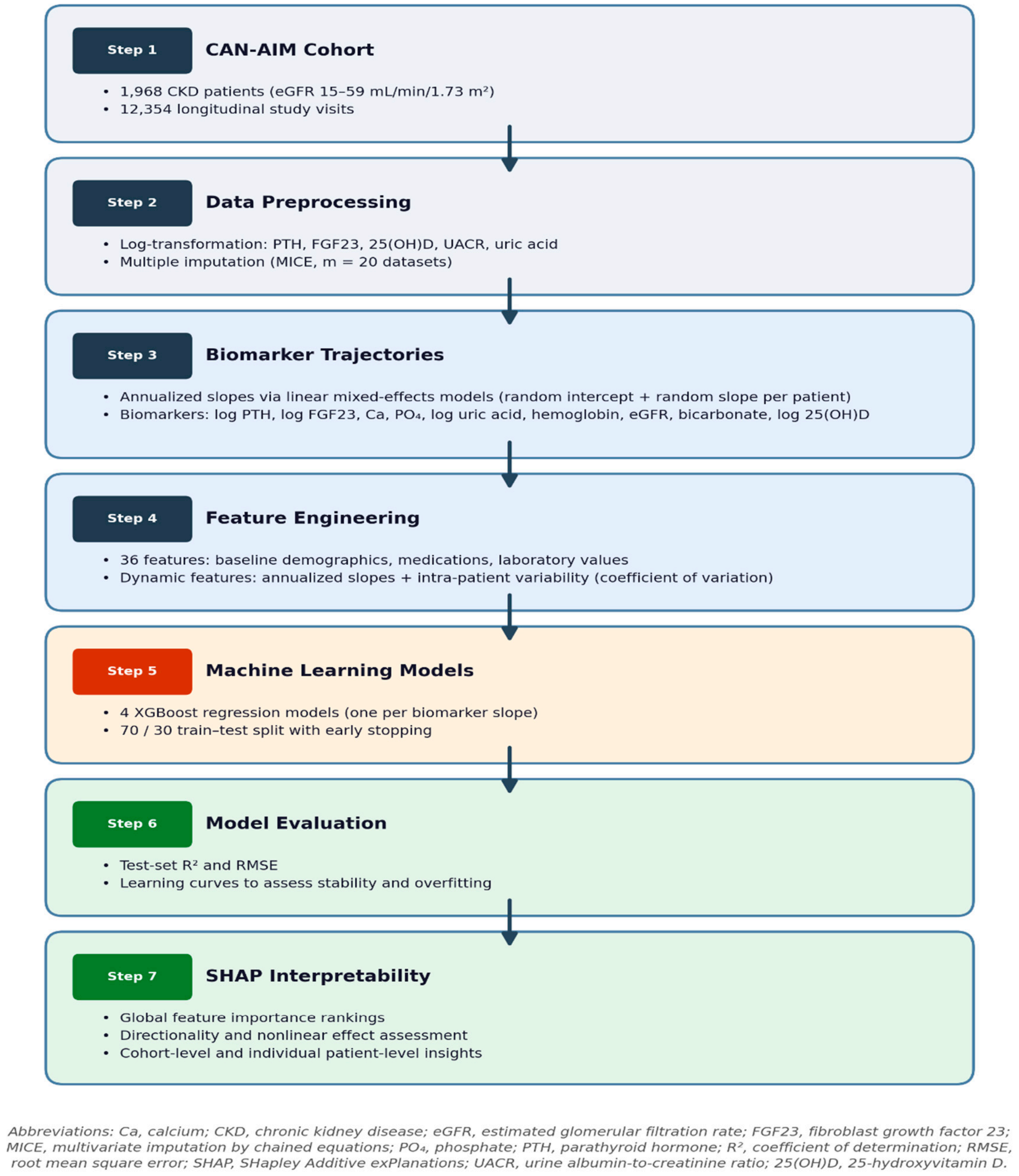

Figure S1. Analytic Workflow for Machine Learning Models and SHAP Interpretability in the CAN-AIM Cohort

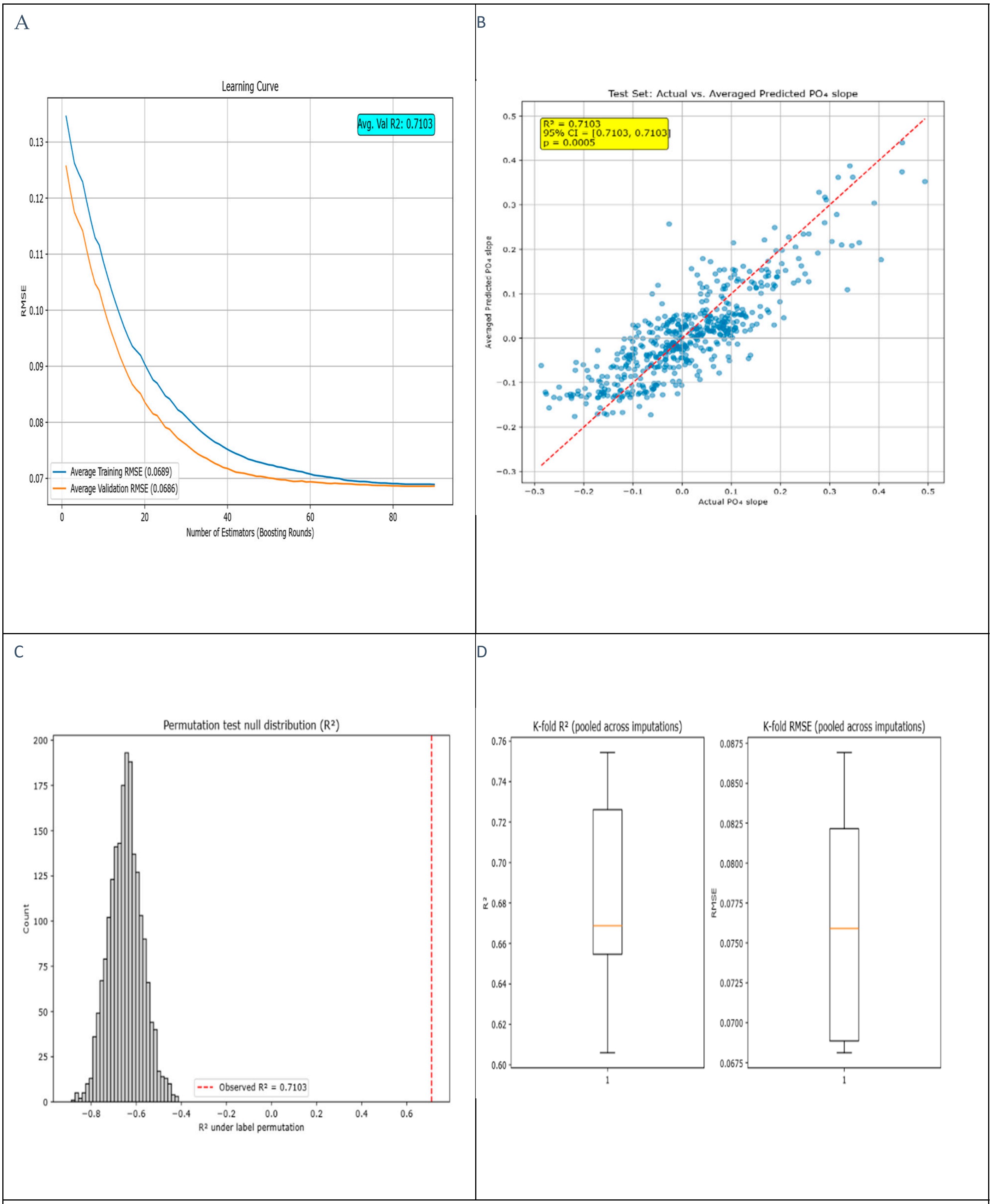

Figure S2. Gradient boosting model performance for predicting phosphate (PO_4) slope; **A.** Learning curve showing synchronized training / validation RMSE; **B.** Predicted vs. observed PO_4 slopes in the test set; **C.** Permutation test null distribution confirming statistical significance; **D.** Distribution of R^2 and RMSE across cross-validation folds and imputations. Abbreviations: R^2: coefficient of determination; RMSE: root-mean-square error; CI: confidence interval; FGF23: Fibroblast growth factor; PO_4: Phosphate

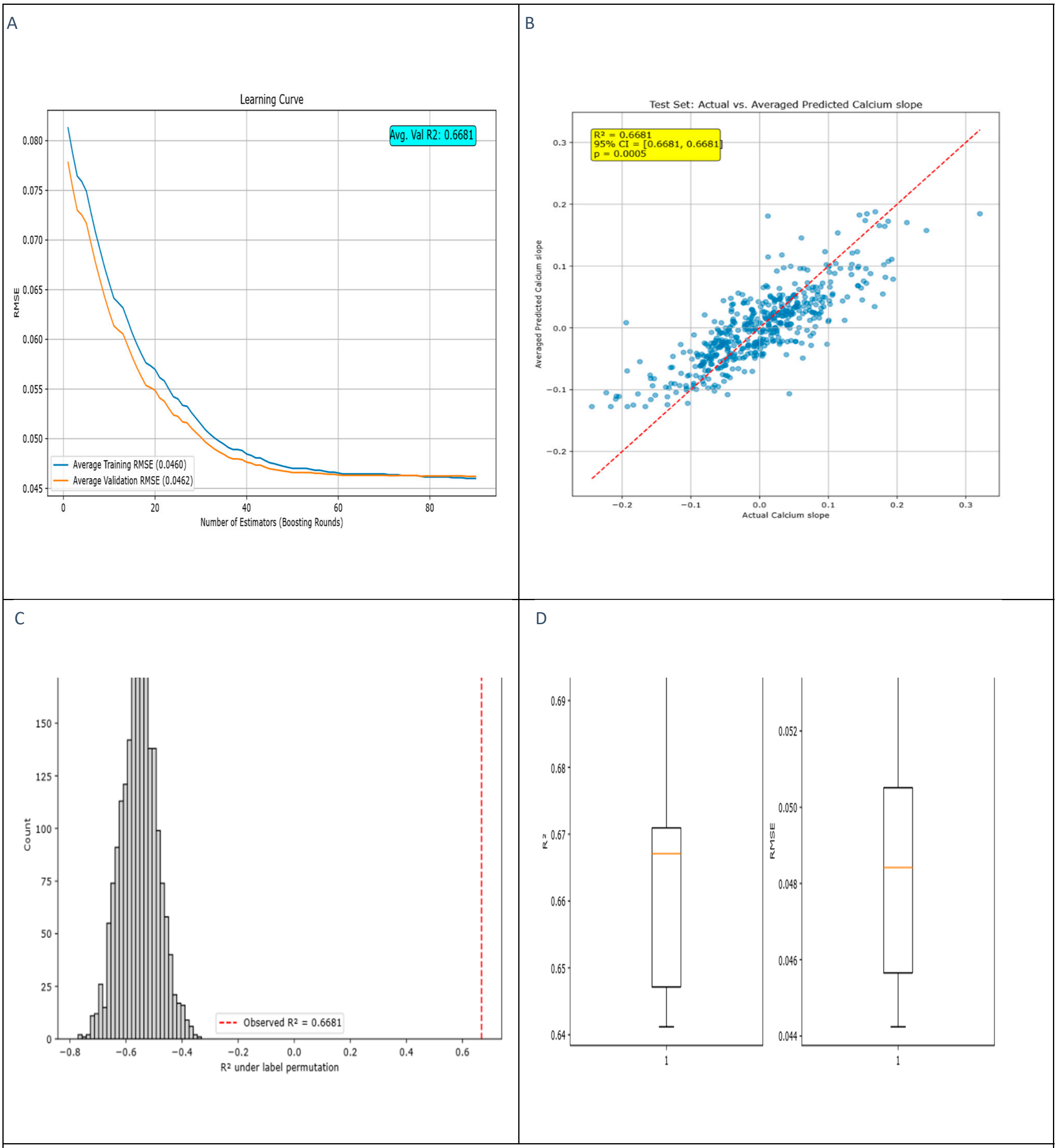

Figure S3. Gradient boosting model performance for predicting calcium slope. A. Learning curve showing training / validation RMSE convergence. **B.** Actual vs. predicted calcium slopes in the test set.**C.** Permutation test null distribution of R^2 confirming significance. **D.** Cross-validated R^2 and RMSE distributions across imputations. Abbreviations: R^2: coefficient of determination; RMSE: root-mean-square error; CI: confidence interval

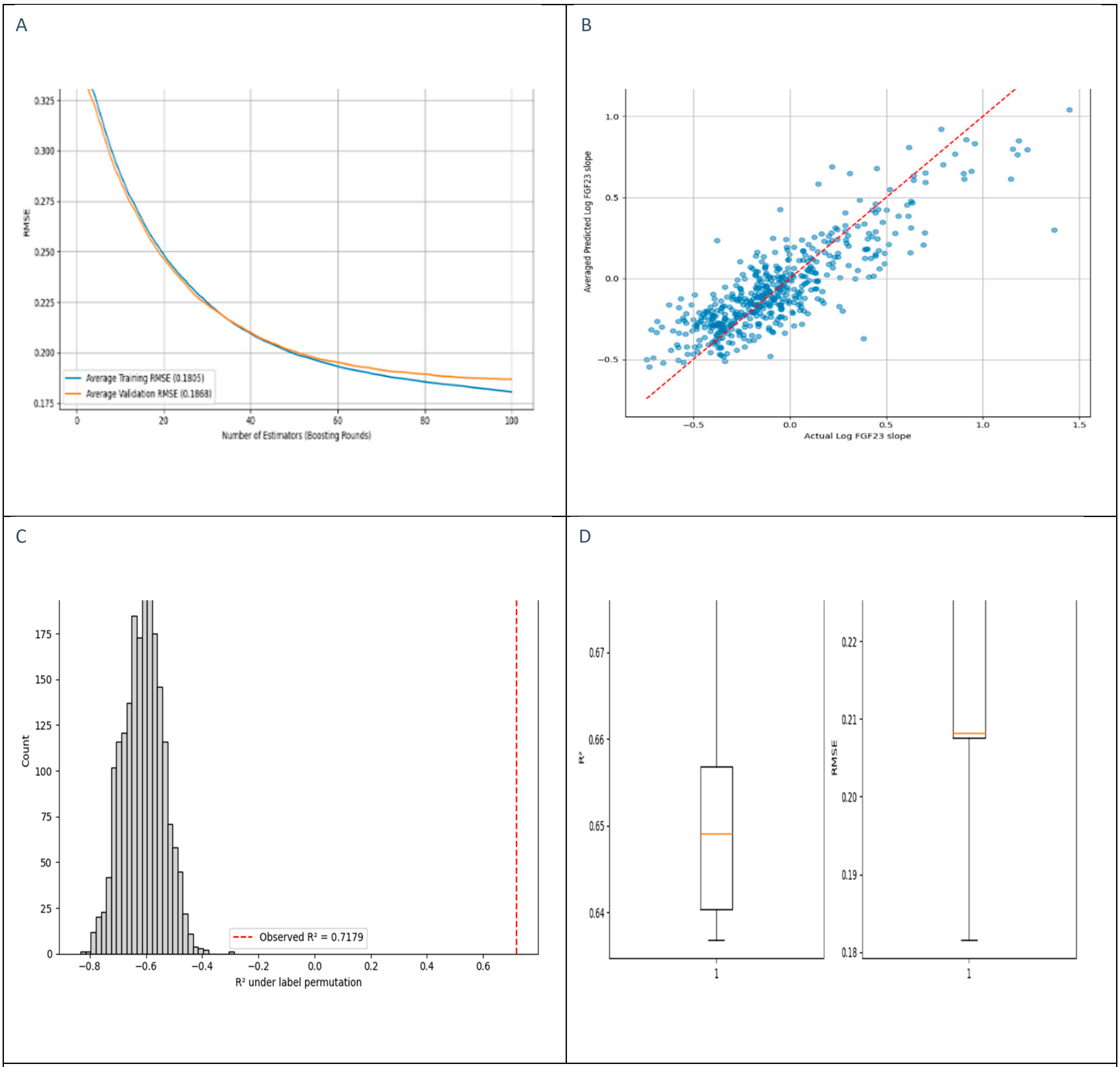

Figure S4. Gradient boosting model performance for predicting log-FGF23 slope.**A.** Learning curve demonstrating stable training / validation convergence. **B.** Observed vs. predicted log-FGF23 slopes in the test set. **C.** Permutation test null distribution verifying model significance. **D.** Fold-wise R^2 and RMSE variability pooled across imputations. Abbreviations: R^2: coefficient of determination; RMSE: root-mean-square error; CI: confidence interval; FGF23: Fibroblast growth factor; log: natural logarithm (base e)

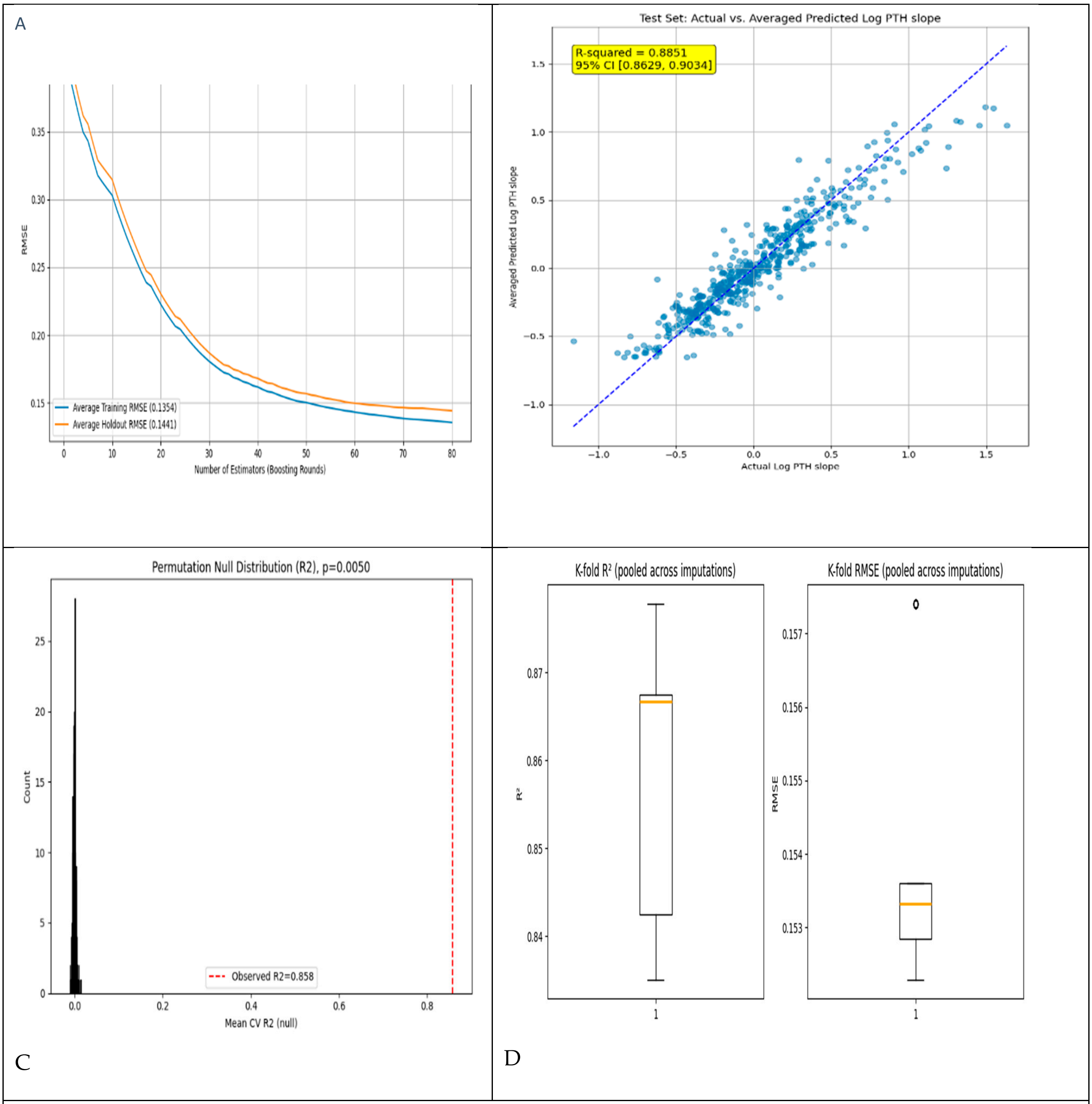

Figure S5. Gradient boosting model performance for predicting log-PTH slope.A. Learning curve demonstrating stable training / validation convergence.**B.** Observed vs. predicted log-PTH slopes in the test set. **C.** Permutation test null distribution verifying model significance. **D.** Fold-wise R^2 and RMSE variability pooled across imputations.Abbreviations: R^2: coefficient of determination; RMSE: root-mean-square error; CI: confidence interval; PTH: Parathyroid hormone; log: natural logarithm (base e)

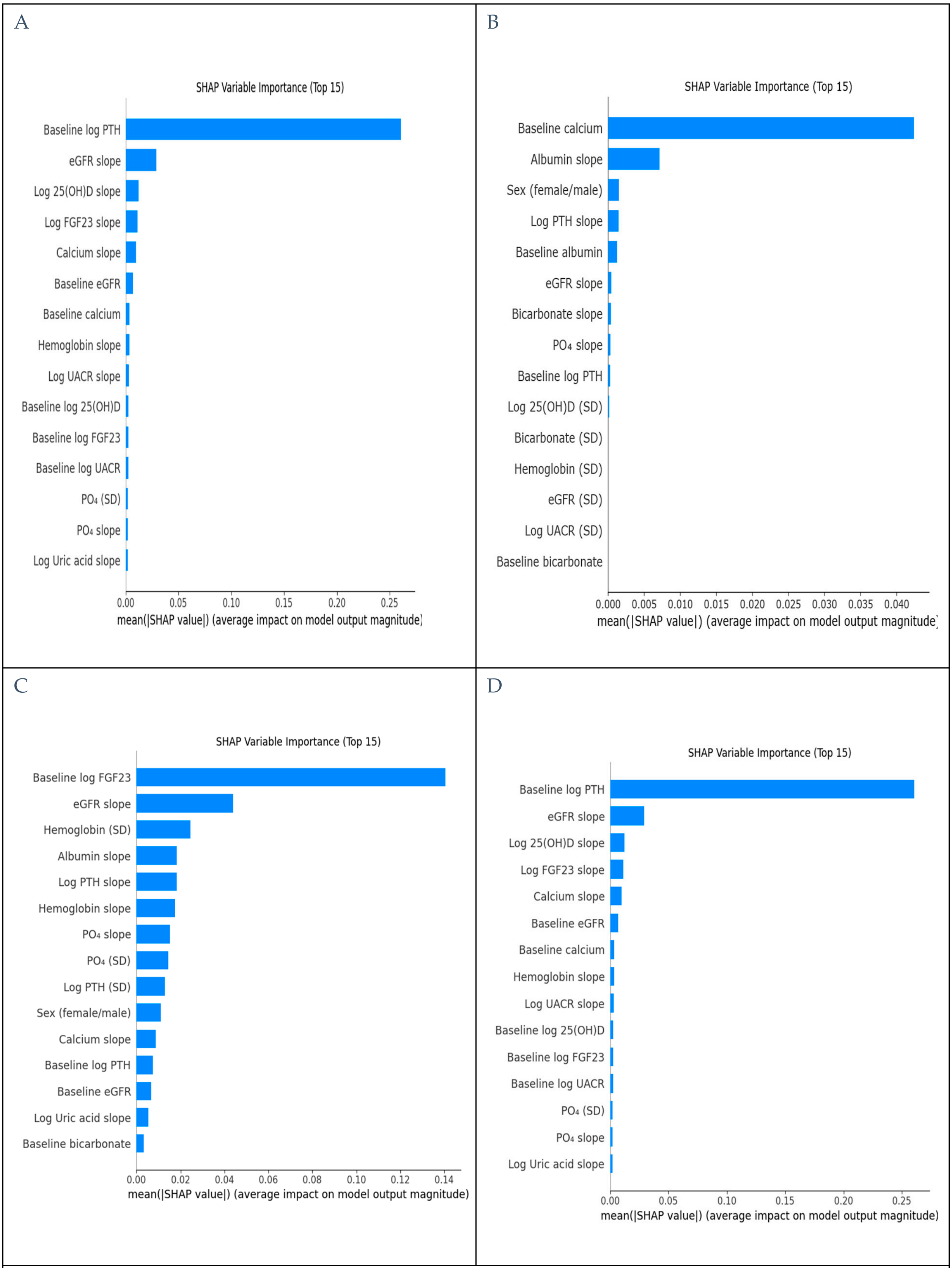

Figure. S6. SHAP bar plot of the top 15 features ranked by mean absolute contribution to the model output **A.** PO4 slope, **B.** Calcium slope, **C.** Log FGF23 slope, **D.** Log PTH slope; Abbreviations: eGFR: estimated glomerular filtration rate; PTH: parathyroid hormone; UACR: urine albumin-to-creatinine

ratio; 25(OH)D: 25-hydroxyvitamin D; PO_4: phosphate; FGF23: fibroblast growth factor-23; SD: standard deviation; log: natural logarithm (base e)
